# Human umbilical cord matrix-derived stem cells exert trophic effects on β-cell survival in diabetic rats and isolated islets

**DOI:** 10.1242/dmm.021857

**Published:** 2015-12-01

**Authors:** Yunting Zhou, Qi Hu, Fuyi Chen, Juan Zhang, Jincheng Guo, Hongwu Wang, Jiang Gu, Lian Ma, Guyu Ho

**Affiliations:** 1 Department of Pediatrics, The Second Affiliated Hospital of Shantou University Medical College, Shantou 515041, China; 2 Department of Molecular Pathology, Shantou University Medical College, Shantou 515041, China; 3 Department of Pediatrics, The Women and Children's Hospital of Shenzhen University, Shenzhen 518122, China

**Keywords:** Stem cells, Diabetes, β-cells, Growth factors, IGF1

## Abstract

Human umbilical cord matrix-derived stem cells (uMSCs), owing to their cellular and procurement advantages compared with mesenchymal stem cells derived from other tissue sources, are in clinical trials to treat type 1 (T1D) and type 2 diabetes (T2D). However, the therapeutic basis remains to be fully understood. The immunomodulatory property of uMSCs could explain the use in treating T1D; however, the mere immune modulation might not be sufficient to support the use in T2D. We thus tested whether uMSCs could exert direct trophic effects on β-cells. Infusion of uMSCs into chemically induced diabetic rats prevented hyperglycemic progression with a parallel preservation of islet size and cellularity, demonstrating the protective effect of uMSCs on β-cells. Mechanistic analyses revealed that uMSCs engrafted long-term in the injured pancreas and the engraftment markedly activated the pancreatic PI3K pathway and its downstream anti-apoptotic machinery. The pro-survival pathway activation was associated with the expression and secretion of β-cell growth factors by uMSCs, among which insulin-like growth factor 1 (IGF1) was highly abundant. To establish the causal relationship between the uMSC-secreted factors and β-cell survival, isolated rat islets were co-cultured with uMSCs in the transwell system. Co-culturing improved the islet viability and insulin secretion. Furthermore, reduction of uMSC-secreted IGF1 via siRNA knockdown diminished the protective effects on islets in the co-culture. Thus, our data support a model whereby uMSCs exert trophic effects on islets by secreting β-cell growth factors such as IGF1. The study reveals a novel therapeutic role of uMSCs and suggests that multiple mechanisms are employed by uMSCs to treat diabetes.

## Abstract (precis)

**Summary:** The newly identified paracrine role of umbilical cord stem cells highlights their therapeutic utilities to treat not only the autoimmune-mediated type 1 diabetes but also type 2 diabetes.

## INTRODUCTION

Diabetes is one of the most prevailing diseases worldwide. It is characterized by hyperglycemia resulting from an absolute or relative insulin deficiency. The chronic hyperglycemia of diabetes is associated with disabling damages of various organs such as the eye, kidneys, nerves and heart. The vast majority of cases of diabetes are type 1 diabetes (T1D) or type 2 diabetes (T2D), accounting for 5-10% and ∼90-95%, respectively (American Diabetes Association, 2014). All T1D and one-third of T2D are caused by substantial deficits in insulin-producing β-cells. Insulin administration is the principal treatment for these insulin-dependent diabetic patients. However, insulin given exogenously does not exactly mimic its physiological secretion and carries the risk of the patient developing hypoglycemia or secondary diabetic complications. Replenishment of deficient β-cells through islet transplantation offers ideal therapeutic outcomes, yet the option is limited by the donor availability, graft survival and complications associated with the long-term use of immunosuppressants (Bromberg et al., 2007; Gruessner and Gruessner, 2013). Exploiting the regenerative capacity of stem cells has thus emerged as a novel approach to complement the current therapies.

Adult mesenchymal stem cells (MSCs), owing to their multipotent and modulatory properties, are intensely used in regenerative medicine (Volarevic et al., 2011). MSCs were first isolated from the bone marrow and have since been found in almost all postnatal tissues. Bone-marrow-derived MSCs (bMSCs) remain as the gold standard in clinical applications. However, bMSCs have specific problems that limit their use. For instance, bMSCs are present at a low frequency in the marrow compartment; the cell-harvesting procedure is painful and invasive; and bMSCs exhibit a reduced *ex vivo* expansion capacity as the donor age increases (Batsali et al., 2013; Watson et al., 2015). On the other hand, human uMSCs can be obtained in large quantities; the cell harvesting is non-invasive; uMSCs are primitive and highly expandable (Troyer and Weiss, 2008); and they can undergo freeze-and-thaw for convenient off-the-shelf use. Studies have also shown that uMSCs do not spontaneously transform in culture (Tang et al., 2013) or form teratomas upon transplantation (Troyer and Weiss, 2008). These cellular features and procurement advantages make uMSCs a promising cell source in cell-based therapies.

Human uMSCs are currently under clinical investigations to treat diabetes (Berezin, 2014). Although the preliminary data from both T1D and T2D studies are promising (Hu et al., 2013; Kong et al., 2014), the mode of action remains to be understood. Because there is little evidence to indicate that MSCs are capable of differentiating into insulin-producing cells *in vivo* (Hess et al., 2003; Lechner et al., 2004; Lee et al., 2006; Taneera et al., 2006), the contribution of differentiation to the treatment effect is likely minimal. MSCs possess the immunomodulatory activity (Abdi et al., 2008; Nauta and Fibbe, 2007), which is shown to be associated with ameliorating hyperglycemia in autoimmune diabetic mice (Bassi et al., 2012; Ezquer et al., 2012). The immunomodulatory function of MSCs could explain the therapeutic benefits seen in T1D, which is caused by immune-mediated β-cell destruction. Yet the mere immune modulation might not be adequate to explain the efficacy seen in T2D, where the β-cell death is chiefly caused by glucotoxicity (Bensellam et al., 2012). It seems that additional mechanisms underlie the therapeutic effect of uMSCs in the treatment of diabetes.

**TRANSLATIONAL IMPACT**

**Clinical issue**

Diabetes is one of the most prevailing diseases worldwide. All cases of type I and one-third of cases of type 2 diabetes are caused by deficits in insulin-producing β-cells. The standard of care for these patients is insulin injection. Yet the treatment has many drawbacks. For instance, patients can experience life-threatening hypoglycemia and many develop disabling diabetic complications. Stem cells, owing to their capacity to differentiate into replacement cells and repair damaged tissues, have emerged as innovative therapies to complement current treatment options. Mesenchymal stem cells from the human umbilical cord matrix (uMSCs) have already shown clinical promise for the treatment of diabetes. However, given that there is little evidence that uMSCs can differentiate into insulin-producing cells *in vivo*, the mechanisms that underlie this promise are not fully understood.

**Results**

Here, the authors use a chemical agent that specifically destroys β-cells to generate diabetic rats and show that infusion of uMSCs into these diabetic rats prevents hyperglycemia by improving insulin secretion and increasing islet cell numbers (85% of islet cells are β-cells). Then, using *in vivo* cell tracking, morphological and biochemical techniques, they show that uMSCs engraft in the chemically injured pancreas and secrete abundant β-cell growth factors, including IGF1. They subsequently show that uMSC engraftment activates the PI3K signaling pathway, which suggests that growth factors secreted by the uMSCs might be the mediators between the protective effect of uMSCs and β-cell survival. To support this assumption, they show that uMSCs promote islet cell survival and insulin secretion in an *in vitro* islet and uMSC co-culture model, and that reduction of IGF1 secretion from uMSCs using RNA interference diminishes the protective effects of uMSCs on islets.

**Implications and future directions**

Human uMSCs are hypoimmunogenic – they either lack, or express low levels of, cell surface molecules capable of stimulating an immune response. In addition, uMSCs suppress lymphocyte activation and induce immune tolerance. These immunosuppressive properties of uMSCs are thought to underlie the therapeutic basis of uMSCs in the treatment of diabetes. The current findings demonstrate, however, that uMSCs can also exert direct protection against β-cell death by secreting β-cell growth factors such as IGF1. Thus, this study identifies a new therapeutic role for uMSCs and suggests that uMSCs might operate through multiple mechanisms to treat diabetes.

MSCs secret a broad spectrum of growth factors and extracellular matrix (ECM) molecules that are increasingly thought to be central to the tissue repair (Baraniak and McDevitt, 2010). These biomolecules can act in a paracrine fashion to promote angiogenesis and ECM remodeling (Baraniak and McDevitt, 2010). Neovascularization has been observed in the damaged pancreas after bMSC transplantation (Ito et al., 2010; Rosengren et al., 2009). Here, we tested whether uMSCs exerted direct trophic effects on β-cells by using the chemically induced diabetes model as well as the *ex vivo* islet and uMSC co-culture system.

## RESULTS

### Systemic administration of uMSCs prevents hyperglycemic progression and body weight loss of STZ-induced diabetic rats

**Fig. 1.**
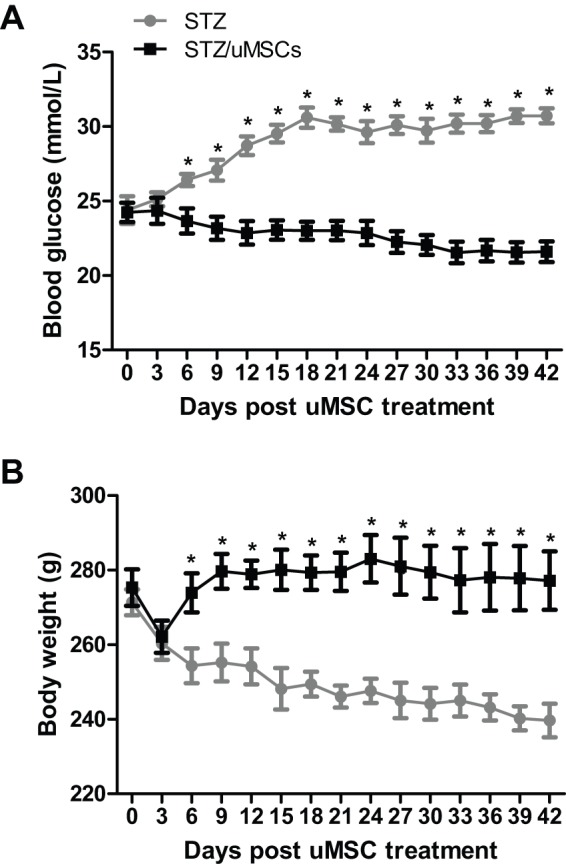
**Effects of uMSC transplantation on hyperglycemia and body weight of STZ-induced diabetic rats.** STZ-treated rats with overt hyperglycemia were injected with either uMSCs or vehicle and the blood glucose level (A) and body weight (B) were measured every 3 days for 42 days. Asterisks (*) denote statistical differences (*P*<0.05) between the uMSC-transplanted and untransplanted rats (*n*=8/group).

The diabetic rat model was established by a single-dose injection of streptozotocin (STZ), which specifically destroys β-cells (Lenzen, 2008). The STZ administration resulted in a rise in blood glucose from euglycemic 6.06 mmol/l to 24.24 mmol/l in 7 days, representing a fourfold increase. Human uMSCs were then systemically administered (day 0). The blood glucose of untransplanted rats continued to rise, reaching 30.60 mmol/l at day 18, but stabilized thereafter (Fig. 1A). The uMSC transplantation retarded hyperglycemic progression at day 6, with the blood glucose becoming modestly lower thereafter (Fig. 1A). The cell treatment also prevented the body weight loss (Fig. 1B). The findings support our previous observation that uMSCs offer therapeutic benefits to diabetic rats (Wang et al., 2014).

**Fig. 2.**
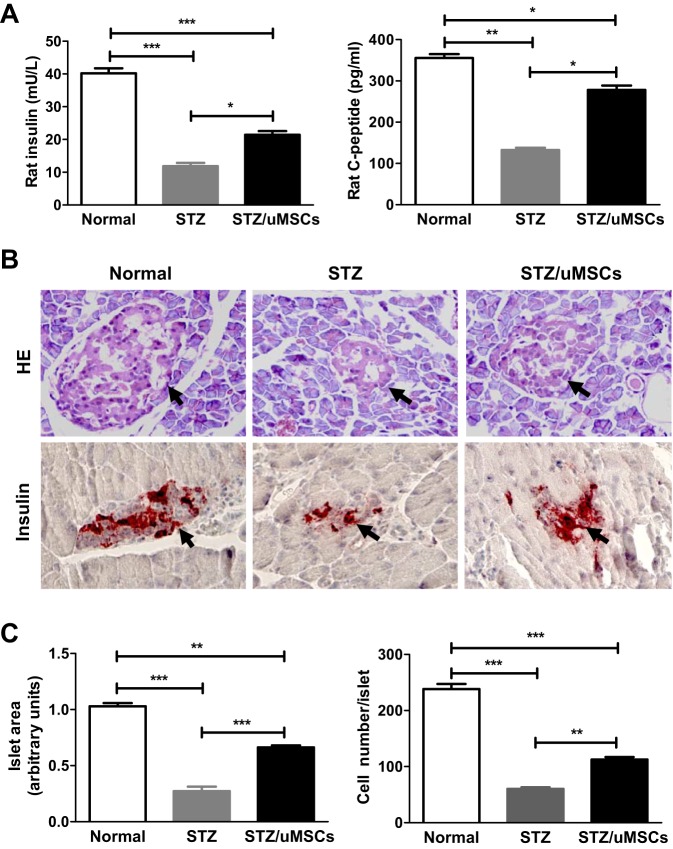
**Effects of uMSC transplantation on islet function and morphology of STZ-induced diabetic rats.** uMSC-transplanted and untransplanted rats were euthanized at 42 days. The rat insulin and C-peptide levels in the serum were measured using the rat-specific ELISA (A). The islet morphology was examined by HE staining and insulin content in the islet was examined by insulin immunohistochemistry; the representative photomicrographs are shown and islets are indicated by black arrows (B). The islet area and cellularity were quantified as described in the Materials and Methods (C). Magnification was at the 40× objective field. *P<0.05; **P<0.01; ***P<0.001. *n*=8/group.

The rat-specific insulin and C-peptide in the serum were measured at day 42 after transplantation. Both parameters were doubled in value after the cell treatment but neither was fully restored, with insulin at 53% and C-peptide at 79% of normal controls (Fig. 2A). Histological analysis showed that untransplanted rats had a markedly reduced islet size and cell number (Fig. 2B); uMSC transplantation partially restored the morphology, with the islet size at 63% and cell number at 42% of normal controls (Fig. 2C). Immunohistochemical analysis further supported the improved islet mass and insulin secretion in transplanted rats (Fig. 2B). Consistent with our previous report (Wang et al., 2014), expression of the human insulin gene was undetectable in the transplanted rat pancreas (data not shown). The data demonstrate that the therapeutic benefits conferred by uSMC transplantation resulted from the preservation/restoration of islet morphology and function.

### Human uMSCs home to the injured pancreas and express genes and proteins of β-cell growth factors

**Fig. 3.**
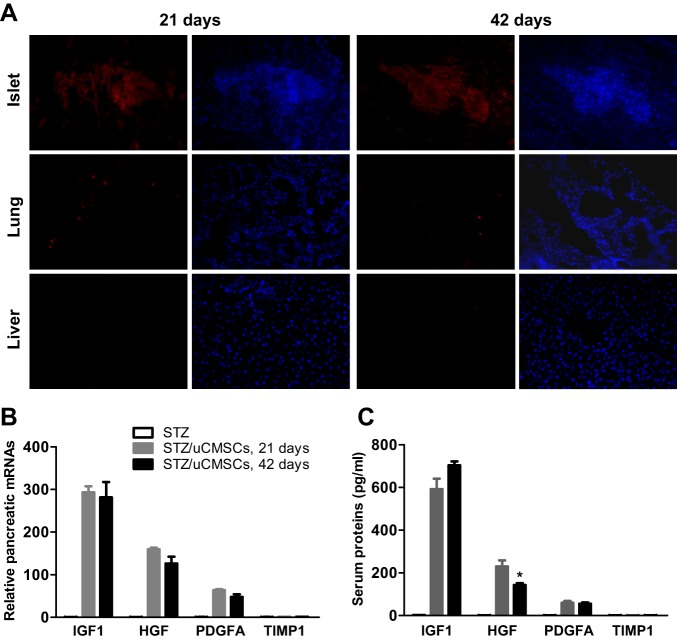
**uMSCs engraft and express genes and proteins of β-cell growth factors in diabetic rats.** (A) Rats transplanted with CM-Dil-labeled uMSCs were euthanized at 21 and 42 days. Sections of the pancreas, lung and liver were examined for CM-Dil fluorescence (red) and the cell population in the section was examined by DAPI staining (blue). Representative photomicrographs with the 40× objective field are shown. (B) Pancreatic mRNA levels of the indicated human genes from transplanted and untransplanted rats (*n*=6/group) were assessed at 21 and 42 days by qPCR using the human-specific primers with GAPDH as internal controls. (C) The corresponding human proteins in the serum of rats (*n*=6/group) were assessed using the human-specific ELISA kits. *P<0.05 was compared with the 21-day transplanted group.

One of the MSC characteristics is the ability to preferentially home to injured tissues upon systemic administration (Deak et al., 2010). To ascertain the pancreatic localization of uMSCs in the diabetic rats, uMSCs were labeled with the fluorescent dye CM-Dil. Studies have shown that CM-Dil does not affect cell viability, proliferation or differentiation (Weir et al., 2008). The *in vitro* cell labeling efficiency was >90% and labeled uMSCs were administered to the diabetic rats. The pancreas as well as the lung and liver, which are rich in blood supply, were examined at 21 and 42 days after the cell infusion. The CM-Dil fluorescence was found clustering around damaged islets at both time points (Fig. 3A). In contrast, little or no fluorescence was found in the lung or liver (Fig. 3A). Infusion of labeled uMSCs ameliorated hyperglycemia and body weight loss of diabetic rats to a similar degree as with unlabeled uMSCs (data not shown), confirming that the labeling did not affect uMSC function.

Because of the pancreatic localization of uMSCs, the pancreas of transplanted rats was screened for expression of human-specific genes of insulin-like growth factor 1 (IGF1), hepatocyte growth factor (HGF) and platelet-derived growth factor alpha (PDGFA) by qPCR. These growth factors, with the cognate receptors expressed on β-cells, have been shown to stimulate β-cell expansion and protect against STZ-induced β-cell death in mice (Chen et al., 2011; Dai et al., 2005; Garcia-Ocana et al., 2000; George et al., 2002). In addition, we examined the expression of tissue inhibitor of metalloproteinase 1 (TIMP1), which promotes β-cell survival under inflammatory conditions and is highly produced by human adipose-derived MSCs (aMSCs) (Kono et al., 2014). The mRNAs of human *IGF1*, *HGF* and *PDGFA* were detected at both 21 and 42 days after the cell transplantation, with the *IGF1* mRNA level being the highest (Fig. 3B). Assessment of the protein secretion in the serum using the human-specific ELISA showed substantial levels at both time points, with the serum levels of human IGF1, HGF and PDGFA at 704, 142 and 55 pg/ml, respectively, at day 42 (Fig. 3C). These factors showed little change at either the mRNA or protein levels between the two time points (Fig. 3B,C). The assay specificities were confirmed by undetectable levels of corresponding human mRNAs or proteins in the untransplanted rat (Fig. 3B,C). Neither the mRNA nor protein of human TIMP1 was detected in transplanted rats (Fig. 3B,C).

### Human uMSCs activate the PI3K pathway in the injured pancreas

**Fig. 4.**
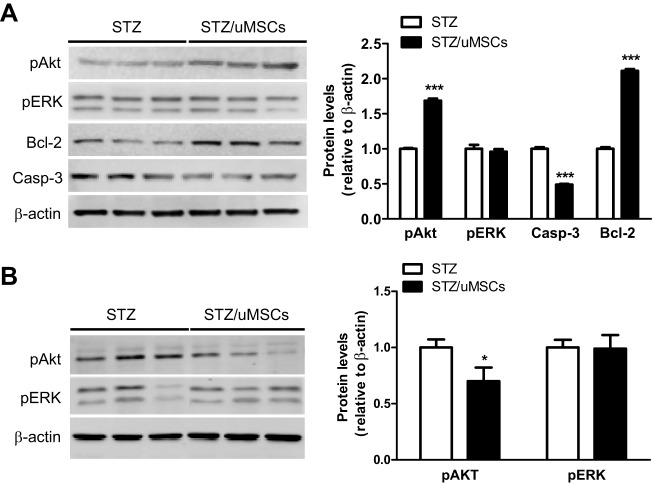
**Assessment of pathway activation in the pancreas and liver of uMSC-transplanted rats.** Pancreatic or liver tissues from the uMSC-transplanted and untransplanted rats (*n*=6/group) were collected at 42 days. (A) Levels of pAkt, pERK1/2, Bcl-2 and caspse-3 in the pancreas were analyzed by western blots and quantification was normalized against β-actin. (B) The liver pAkt and pERK1/2 were assessed as in A. *P<0.05 and ***P<0.001 were compared with the STZ group.

IGF1, HGF and PDGFA are shown to activate the PI3K and ERK1/2 pathways in isolated islets or β-cell lines (Chen et al., 2011, 2014; Dai et al., 2005). The abundant production of the growth factors by uMSCs prompted us to assess the activation state of the two signaling pathways in the pancreas. Shown in Fig. 4A, a marked Akt, but not ERK1/2, phosphorylation was seen in the transplanted pancreas. To rule out the contribution of human pAkt owing to the uMSC engraftment, we assessed the relative mRNA levels between the rat and human GAPDHs in the pancreas. The ratio, as evaluated by qPCR, was >1000. Thus, the human pAkt, if any, was negligible in the pAkt level observed. Assessment of the downstream anti- and pro-apoptotic effectors of Bcl-2 and caspase-3 showed a 2.1-fold elevation and 52% reduction, respectively, in transplanted compared with untransplanted rats (Fig. 4A). The data demonstrate that uMSC transplantation activated the pancreatic PI3K pathway with subsequent activation of the anti-apoptotic machinery. Because of high systemic levels of the growth factors, especially uMSC-secreted IGF1, we evaluated the impact on pAkt and pERK1/2 levels in the liver. In contrast to the pancreas, the liver pAkt exhibited a modest reduction in transplanted rats as compared with the untransplanted control (Fig. 4B). The liver pERK1/2 level did not differ between the two groups (Fig. 4B).

### Human uMSCs improve the viability and function of rat islets in co-culture

**Fig. 5.**
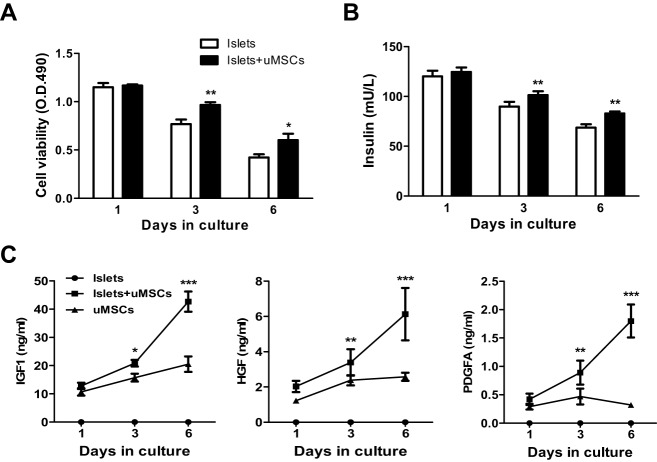
**Assessment of the rat islet survival/function and uMSC-secreted growth factors in culture.** Freshly isolated rat islets were cultured alone or with uMSCs for the indicated time points. The islet cell viability was measured using the MTS assay (A) and the rat insulin level in supernatants was measured using the rat-specific ELISA (B). Supernatants from the uMSC or islet-alone culture or the co-culture were taken at the indicated times, and levels of human IGF1, HGF and PDGFA were measured using the human-specific ELISA kits (C). The data are representatives of at least three independent experiments. *P<0.05, **P<0.01 and ***P<0.001 indicate the statistical significance between the uMSC and islet/uMSC cultures.

To support the direct trophic effect of uMSCs on islets (containing 85% β-cells) and that the secreted factors mediated the effect, uMSCs were co-cultured with isolated rat islets in transwell plates, in which only soluble factors were freely passable between the two cell compartments. The islet culture alone exhibited a progressive cell death; the cell viability at day 6 was 37% of that of day 1 but was improved by 42% of this day-6 value in the co-culture (Fig. 5A). Similarly, the islet culture alone exhibited a progressive decline in insulin secretion; the insulin level at day 6 was 57% of that of day 1 and was enhanced by 21% in the co-culture (Fig. 5B). The data demonstrate that uMSCs exerted direct pro-survival effects on islets.

To assess the secretion of IGF1, HGF and PDGFA by uMSCs in culture, uMSCs were cultured alone or co-cultured with islets and the supernatants were taken for the measurement using the human-specific ELISA. As shown in Fig. 5C, these factors were produced with the same rank order as that observed *in vivo*, with IGF1 being the highest. Notably, their productions were augmented by co-culturing with islets. The IGF1 level in the co-culture at day 6 was 43 ng/ml, which was sevenfold higher than HGF and 24-fold higher than PDGFA (Fig. 5C). The assay specificity was again confirmed by undetectable levels of the human proteins in the rat islet culture alone (Fig. 5C). The TIMP1 production by uMSCs in culture was also undetectable (data not shown).

### The uMSC-secreted IGF1 promotes the islet cell survival and function

**Fig. 6.**
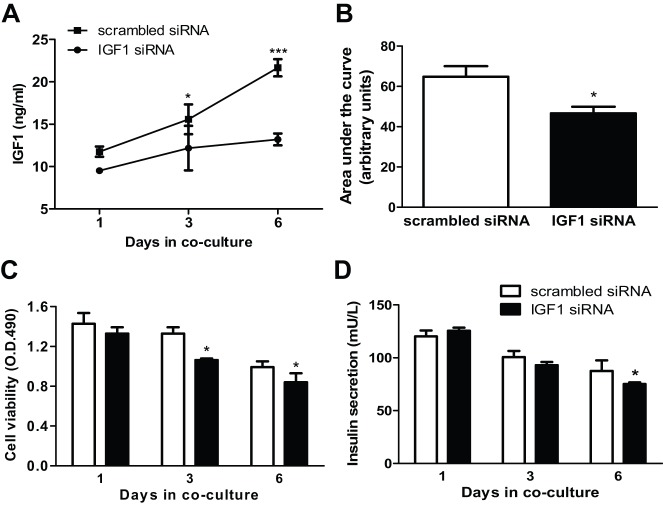
**Effects of reduced IGF1 secretion by uMSCs on islet survival and function in co-culture.** uMSCs transfected with either the IGF1-specific or scrambled siRNA were co-cultured with islets for up to 6 days. The human IGF1 levels in supernatants were measured at the indicated time points (A) and the area under the curve of the human IGF1 production in the 6-day culture was calculated (B). In parallel experiments, the islet survival (C) and insulin secretion (D) were measured. The data are representatives of at least three independent experiments. *P<0.05 and ***P<0.001 indicate the statistical significance between the scrambled and *IGF1* siRNA cultures.

Because of the abundant production of human IGF1 both *in vivo* and *in vitro*, we investigated the contribution of uMSC-secreted IGF1 to islet viability and function by using siRNA knockdown. Our preliminary studies showed that transfection of the *IGF1*-specific siRNA to uMSCs gave the best protein knockdown at 48 h (data not shown). Thus, uMSCs transfected for 48 h with either the specific or scrambled siRNA were utilized in the co-culture studies. As shown in Fig. 6A, the IGF1 production was persistently reduced by the *IGF1* siRNA in the 6-day co-culture. The area under the curve of the IGF1 production in the 6-day specific knockdown co-culture was reduced by 27% as compared with the scrambled control (Fig. 6B). In parallel assays, the islet viability was diminished in both 3-day and 6-day co-cultures, with a 17% reduction in the 6-day co-culture (Fig. 6C); the insulin secretion was reduced by 14% in the 6-day co-culture (Fig. 6D). Thus, the data establish the crucial contribution of uMSC-secreted IGF1 to islet survival and function.

## DISCUSSION

In this study, the diabetic model was generated by a single large-dose injection of STZ. Under such conditions, STZ destroys β-cells via DNA alkylation (Lenzen, 2008). The model differs from the autoimmune diabetic model of non-obese diabetes (NOD) or multiple low-dose STZ-induced diabetes, where islet inflammation is the salient pathological feature (Bassi et al., 2012; Ezquer et al., 2012; George et al., 2002; Leiter, 1982; Paik et al., 1980). The non-inflammatory diabetic model used here, which was confirmed by the lack of inflammatory cell infiltrates in the islet of untransplanted but STZ-treated rats (Fig. 2B), allows us to better examine the direct trophic effect of uMSCs on β-cell survival with fewer complications from their immunomodulatory activities. In this study, uSMC transplantation retarded the hyperglycemic progression 6 days after the treatment, yet the treatment only modestly reversed hyperglycemia despite the extended 42-day treatment. Multiple factors might influence the efficacies. Because the transplanted cells were apparently alive and functional for the entire 42 days (discussed below), we suspect that the delayed treatment intervention (i.e. the glucose level was already fourfold higher than normal) limited the efficacy. An earlier intervention with more residual islets left for regeneration might produce better therapeutic outcomes.

Human uMSCs were found localized to the injured pancreas but not the lung or liver. The finding concurs with the general homing characteristics of MSCs to injured tissues (Deak et al., 2010) and the homing pattern of human bMSCs in diabetic mice (Lee et al., 2006). Notably, the xenogeneic graft survived long-term in the absence of immunosuppressants. The successful engraftment was further supported by the stable expression of human genes in the rat pancreas. Because of the lack of inflammatory infiltrates in and around the islet of transplanted rats (Fig. 2), uMSCs did not seem to have provoked the host immune response. Our data support the long-held notion that uMSCs are hypoimmunogenic (Weiss et al., 2008, 2006), which, coupled with the immunosuppressive property (McGuirk and Weiss, 2011), might have contributed to the muted graft rejection. Studies have reported that transplantation of human uMSCs in immunocompetent rats results in functional improvements of damaged organs (Lopez et al., 2013; Weiss et al., 2006); however, the cell fate *in vivo* is not clear. The demonstration that uMSCs can survive long-term and remain functional in xenogeneic hosts could have clinical implications. For instance, it might help project the cell treatment frequency and suggest a reduced or even eliminated immunosuppressant use in the allogeneic cell therapies.

Consistent with the stable mRNA expression in the pancreas, human IGF1, HGF and PDGFA proteins were stably detected in the serum of transplanted rats, among which IGF1 was most abundant. The local concentrations in the pancreas, where the proteins were produced, could be even higher and might thus act effectively as the β-cell trophic factors. In support, a marked activation of the pancreatic PI3K pathway and downstream anti-apoptotic machinery was evident. We also analyzed the pro-growth potential of the PI3K activation by assessing the downstream cell-cycle genes such as cyclin D1, cyclin D2, p21 and p27. Expression of these genes is involved in β-cell proliferation (Heit et al., 2006). We saw ∼twofold upregulation of the cyclin D2 mRNA level but expression of the other genes was unchanged in the pancreas of transplanted rats (data not shown). The limited expression changes of cell-cycle genes imply that the β-cell expansion was restrained, which agrees with the modest reversal of hyperglycemia observed. These biochemical data suggest that the anti-apoptotic activity of uMSCs is the main contributor to the improved islet size found in the histological study. Despite the marked activation of the pancreatic PI3K pathway, we did not see pancreatic ERK1/2 activation. We suspect that, in contrast to the fundamental role of PI3K in β-cell survival (Bernal-Mizrachi et al., 2001; Tuttle et al., 2001), the role of ERK might be auxiliary and that a modest activation in β-cells could be masked by the whole-tissue extracts used in the analysis.

The siRNA knockdown experiment showed that a 27% reduction in uMSC-secreted IGF1 led to a 17% reduction of β-cell survival. Although the study illustrates the major role of uMSC-secreted IGF1, the disproportionate reduction implies that other β-cell trophic factors, although playing minor roles, are required to fully support the β-cell survival. The uMSC-secreted HGF and PDGFA, both produced in the nM range in co-cultures and detectable in transplanted rats, could be the other possible mediators. It is worthy of noting that secretion of these factors by uMSCs was induced by islet cells. An augmentation of aMSC-secreted factors by islet cells has also been observed (Kono et al., 2014). Although the molecular mechanism underlying the cross-talk between uMSCs and islets remains to be elucidated, the induced production by islets, in conjunction with the long-term survival of uMSCs in the injured pancreas, could argue in favor of cell-based over factor-based strategies in diabetes treatment (Baraniak and McDevitt, 2010).

The β-cell growth factors secreted by uMSCs are pleotrophic (Clemmons and Maile, 2003; Nakamura et al., 2011; Ronnstrand, 2004). Whether their systemic exposure leads to undesired effects should be of clinical concern. Transplantation of uMSCs reduced the pAkt level and had no effect on pERK1/2 in the liver of transplanted rats, which might suggest a low potential in the proliferative growth of normal tissues. IGF1 is noted to act mainly on injured tissues (Agudo et al., 2008). Nevertheless, the liability should be assessed over a longer term. We should also point out that these growth factors might play multiple roles in pancreas repair. For instance, PDGFA is also a potent angiogenic factor (Shibuya, 2013) and angiogenesis could be important for islet regeneration (Ito et al., 2010; Rosengren et al., 2009). IGF1 has recently being shown to stimulate regulatory T (Treg) cells and suppress autoimmune diabetes in mice (Anguela et al., 2013; Bilbao et al., 2014). In the study, the recombinant human IGF1 delivered by minipumps to maintain a serum level of ∼350 pg/ml is efficacious in the treatment of diabetes (Bilbao et al., 2014). Using this dose as a gauge, IGF1 stably produced by uMSCs with the serum level of 704 pg/ml and possibly higher levels in the pancreas might exert dual trophic and immunomodulatory functions to preserve/regenerate islets. Notably, IGF1 might also contribute to the efficacy by enhancing insulin sensitivity in diabetic individuals (Clemmons, 2012).

In conclusion, we have two key novel findings in this study: (1) uMSCs exert trophic effects on β-cell survival by activating the pancreatic PI3K pathway; and (2) the trophic effect is mediated by uMSC-secreted factors, among which IGF1 might play a major role.

## MATERIALS AND METHODS

### Animals

Sprague Dawley (SD) rats were purchased from the Animal Center of Shantou University Medical College. Animals were housed under standard conditions with food and water given *ad libitum*. All experimental procedures were conducted in accordance with the guidelines published in the Ministry of Science and Technology of China for the Care and Use of Laboratory Animals and approved by the Animal Care and Welfare Committee of Shantou University Medical College.

### Isolation and expansion of uMSCs

Protocols for obtaining human umbilical cords were reviewed and approved by the Medical Ethics Committee of Shantou University Medical College (SUMC2013XM-0036). The umbilical cord donors gave informed consent. Isolation of uMSCs was carried out as described (Tang et al., 2013). Isolated uMSCs were cultured at 37°C with 5% CO_2_ in growth medium containing GlutaMAX™-1, 10% FBS and 1% penicillin-streptomycin (all from Gibco, USA). The cells were expanded for 2-4 passages before use.

### Induction of diabetes and cell transplantation

Eight-week-old SD rats were given the intraperitoneal injection of STZ at 65 mg/kg body weight (Sigma, USA) dissolved in 0.01 M citrate buffer at pH 4.2 or the buffer alone. Blood glucose was measured daily with the Glucotrend glucose meter (Roche Diagnostics, Switzerland) for the first 7 days to confirm the establishment of diabetic conditions. At day 7 after the STZ treatment, rats were administered, via the tail vein, either 3×10^6^ uMSCs in 200 μl PBS or the vehicle. Blood glucose and body weight were measured every 3 days thereafter for 42 days.

### Tissue collection

Rats were euthanized using 10% chloral hydrate at 0.4 ml/100 g body weight at 21 or 42 days after the cell injection. The trunk blood was collected and the serum was obtained and stored at −80°C until use. The pancreas and liver from each rat were quickly removed. A main portion of the pancreas was fixed with 4% paraformaldehyde and embedded in paraffin for histological and immunohistochemical analyses. The remaining pancreas and liver were immediately snap-frozen in liquid nitrogen and stored at −80°C for PCR and western blot analyses.

### *In vivo* cell tracking

Human uMSCs were labeled with CM-Dil according to the manufacturer's instructions (Invitrogen, USA). The labeling efficiency was examined under fluorescent microscopy and the viability of labeled cells was confirmed by their ability to grow *in vitro*. After labeling, cells were washed with PBS and 3×10^6^ cells were injected into the STZ-treated rats as above. Rats were euthanized at the indicated times and paraffin sections (4-µm thickness) of selected tissues were stained with DAPI (Vectashield, USA) for 5 min at room temperature. The CM-Dil and DAPI staining was visualized and acquired by the fluorescent microscopy (Olympus f1000, Japan).

### Immunohistochemistry

Paraffin sections (4-µm thickness) were stained with hematoxylin and eosin (HE) for histological examination. For the immunohistochemical detection of insulin, paraffin sections were incubated with the rabbit anti-rat insulin antibody (cat#4590, Cell Signaling Technology, USA) at 1:100 dilution, followed by incubation with the HRP-conjugated anti-rabbit secondary antibody (cat#PV-9000, ZSGB-BIO, China) and development with the HRP-AEC kit (GENMED Scientifics, USA). Sections were then counterstained with hematoxylin and examined under the light microscope (Leica DM2000, Germany). For measurement of the islet area, islets in HE sections were identified and the islet area was measured using Image-Pro software (Media Cybernetics, USA). The average islet area from each rat was determined from 4-5 sections spaced >200 μm apart. The cell number per islet area in the same section was counted using Image-Pro (Media Cybernetics, USA) and the average cell number per islet from each rat was determined in the same manner as that of the islet area.

### Quantitative PCR

Total RNAs of the pancreas were extracted using TRIzol according to the manufacturer's instructions (Life Technologies, USA). Total RNAs (1 μg) were reverse-transcribed using the Transcriptor First-strand cDNA Synthesis kit (Takara, Japan) and qPCR was performed using the SYBR^®^ Premix Ex Taq™ II kit (Takara, Japan). Human-specific primers for the indicated genes were designed using the GenBank database and are listed in Table 1. Reactions were run in the 7500 Fast Real-Time PCR System (Applied Biosystems, USA) under the following conditions: 95°C for 15 min, followed by 40 cycles of 94°C for 15 s, 55°C for 30 s and 72°C for 30 s. Quantification of relative mRNA levels was performed using the ΔCt method (Livak and Schmittgen, 2001) with the 7500 Software v2.0.6 (Applied Biosystems, USA).

**Table 1.**
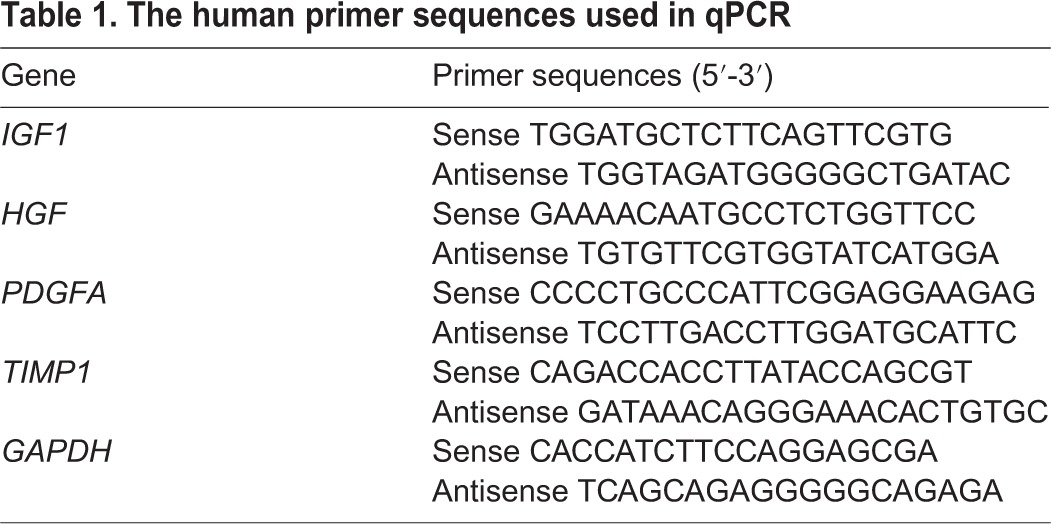
**The human primer sequences used in qPCR**

### Western blot analysis

Proteins were extracted from tissues using the RIPA buffer (Cell Signaling Technology, USA) containing 1 mM NaF and 1 mM phenylmethylsulfonyl fluoride (PMSF; Solarbio, China). Protein concentrations were determined using the BCA assay (Thermo Scientific, USA). Equivalent amounts of protein samples were separated by 10% SDS-PAGE and transferred to nitrocellulose membranes. The primary antibodies used were rabbit anti-phospho-Akt (cat#4060s), rabbit anti-phospho-ERK1/2 (cat#4370s), rabbit anti-caspase-3 (cat#9664) and rabbit anti-Bcl-2 (cat#2870) (all from Cell Signaling Technology, USA; used at 1:1000 dilution) or the mouse anti-β-actin (TA09, ZS-Bio, China; used at 1:2000). The secondary antibodies used were IRDye 680RD-conjugated goat anti-rabbit (cat#926-32220) or IRDye 800CW-conjugated goat anti-mouse (cat#926-32211) antibodies (LI-COR, USA; used at 1:10,000). The band density was scanned using the Odyssey Two-Color Infrared Imaging System (LI-COR, USA) and quantified using the Quantity One System (Bio-Rad, USA).

### Rat islet isolation and co-culture

Rat islets were isolated and purified as described previously (Peng et al., 2009). A total of 70-100 rat islets were used per well. For co-culture experiments, 1×10^5^ uMSCs were seeded into the lower chamber of the 24-well transwell plate (Corning, USA) containing the co-culture medium (i.e. the growth medium with only 5% FBS). Immediately after uMSCs became attached, the medium was changed and freshly isolated islets were placed into the upper chamber. The plates were incubated at 37°C with 5% CO_2_ for the indicated times before the analysis.

### siRNA knockdown

Human uMSCs were seeded into the lower chamber of the 24-well transwell plate and grown to 70-80% confluence. The human-specific *IGF1* siRNA (5′-ACCUUGUCUAAGUGGUUUA-3′) or scrambled siRNA (Gene Pharma, China) was transfected using the Lipofectamine RNAiMAX Reagent according to the manufacturer's instructions (Invitrogen, USA). Transfected uMSCs were cultured in the growth medium for 48 h before the medium was replaced with the co-culture medium. Freshly isolated islets were then placed in the upper chamber for co-culture studies.

### Cell viability and ELISA

Cell viability was measured by the colorimetric MTS assay (absorbance at 490 nm) according to the manufacturer's instructions (Promega, USA). Rat insulin and C-peptide in sera and culture supernatants were measured using the ultrasensitive rat-specific ELISA kit as specified (MeilianBio, China). The 24-h basal insulin secretion in rat islet cultures was assessed as follows: the medium was changed on the day specified and the rat insulin level in the supernatant was measured 24 h later. The levels of human IGF1, HGF and PDGFA in the serum and supernatants were quantified using the human-specific ELISA kits according to the manufacturer's instructions (Abcam, USA).

### Statistical analysis

The Student's *t*-test and one-way ANOVA were used for group comparisons (GraphPad Software Inc., USA). Data were expressed as the mean±standard error of the mean (s.e.m.). *P*<0.05 was considered statistically significant.
